# An ontology-based approach for modelling and querying Alzheimer’s disease data

**DOI:** 10.1186/s12911-023-02211-6

**Published:** 2023-08-08

**Authors:** Francesco Taglino, Fabio Cumbo, Giulia Antognoli, Ivan Arisi, Mara D’Onofrio, Federico Perazzoni, Roger Voyat, Giulia Fiscon, Federica Conte, Marco Canevelli, Giuseppe Bruno, Patrizia Mecocci, Paola Bertolazzi, for the Alzheimer’s Disease Neuroimaging Initiative

**Affiliations:** 1 Institute of Systems Analysis and Computer Science “Antonio Ruberti” (IASI), National Research Council (CNR), Via dei Taurini 19, 00185 Rome, Italy; 2 Genomic Medicine Institute, Lerner Research Institute, Cleveland Clinic, 9500 Euclid Avenue, 44195 Cleveland, Ohio USA; 3 European Brain Research Institute (EBRI) “Rita Levi-Montalcini”, Viale Regina Elena 295, 00161 Rome, Italy; 4 Department of Engineering, Uninettuno International University, Corso Vittorio Emanuele II 39, 00186 Rome, Italy; 5 Department of Engineering, University of Roma Tre, Via della Vasca Navale 79/81, 00146 Rome, Italy; 6 Department of Computer, Control, and Management Engineering “Antonio Ruberti”, Sapienza University of Rome, Via Ariosto 25, 00185 Rome, Italy; 7 Department of Human Neuroscience, Sapienza University of Rome, Via Ariosto 25, 00185 Rome, Italy; 8 Department of Medicine and Surgery, University of Perugia, Piazzale Gambuli 1, 06129 Perugia, Italy; 9 Division of Clinical Geriatrics, NVS Department, Karolinska Institutet, Nobels väg 5, Solna, 17177 Stockholm, Sweden

**Keywords:** Alzheimer’s disease, Ontology, Standardization, Interoperability

## Abstract

**Background:**

The recent advances in biotechnology and computer science have led to an ever-increasing availability of public biomedical data distributed in large databases worldwide. However, these data collections are far from being “standardized” so to be harmonized or even integrated, making it impossible to fully exploit the latest machine learning technologies for the analysis of data themselves. Hence, facing this huge flow of biomedical data is a challenging task for researchers and clinicians due to their complexity and high heterogeneity. This is the case of neurodegenerative diseases and the Alzheimer’s Disease (AD) in whose context specialized data collections such as the one by the Alzheimer’s Disease Neuroimaging Initiative (ADNI) are maintained.

**Methods:**

Ontologies are controlled vocabularies that allow the semantics of data and their relationships in a given domain to be represented. They are often exploited to aid knowledge and data management in healthcare research. Computational Ontologies are the result of the combination of data management systems and traditional ontologies. Our approach is i) to define a computational ontology representing a logic-based formal conceptual model of the ADNI data collection and ii) to provide a means for populating the ontology with the actual data in the Alzheimer Disease Neuroimaging Initiative (ADNI). These two components make it possible to semantically query the ADNI database in order to support data extraction in a more intuitive manner.

**Results:**

We developed: i) a detailed computational ontology for clinical multimodal datasets from the ADNI repository in order to simplify the access to these data; ii) a means for populating this ontology with the actual ADNI data. Such computational ontology immediately makes it possible to facilitate complex queries to the ADNI files, obtaining new diagnostic knowledge about Alzheimer’s disease.

**Conclusions:**

The proposed ontology will improve the access to the ADNI dataset, allowing queries to extract multivariate datasets to perform multidimensional and longitudinal statistical analyses. Moreover, the proposed ontology can be a candidate for supporting the design and implementation of new information systems for the collection and management of AD data and metadata, and for being a reference point for harmonizing or integrating data residing in different sources.

**Supplementary Information:**

The online version contains supplementary material available at 10.1186/s12911-023-02211-6.

## Background

The advances in technology and communications of the last decades have allowed biomedical data (clinical, imaging, multi-omics) to be generated in a massive manner and to be distributed in different databases. This huge amount of data is an immense resource for researchers, even if largely unexploited. Nowadays, one of the most important challenges for the bioinformatics community is to find effective solutions for the management, analysis, and integration of these data. This happens in the context of neurodegenerative diseases, for which several international projects have been raised with the aim at collecting data for the diagnosis, prevention, and treatment of these serious pathologies (see AD data collections and ontologies section for an in-depth review of the main initiatives on these diseases, especially on Alzheimer’s disease).

An increasing number of people are affected by these diseases for which there are not many treatments. Better stratified and earlier diagnoses could target treatments to improve patients’ lives and reduce social and economic costs. So, there is a pressing need to find and validate biomarkers both to predict future clinical decline and to be used as outcome measures in clinical trials of disease-modifying agents, with the ultimate goal to foster the development of innovative drugs.

Machine learning (ML) techniques seem to be the right answer to this need. In fact, (i) an ever-increasing amount of data have been collected on Alzheimer’s disease (AD) during the last decade; (ii) semantic ML techniques have been deeply studied and improved and explainable AI methods and techniques have been investigated to try to obtain causal models even with black box techniques like deep learning [1]; hence user-friendly interfaces are needed to query medical data to obtain multivariate data set for multidimensional statistical analysis.

Considering that, the goal of our work becomes to design and implement a first nucleus of *AD-Onto*, an ontology for Alzheimer’s disease, based on a shared body of attributes, inspired by the Alzheimer Disease Neuroimaging Initiative (ADNI) (https://adni.loni.usc.edu/) data collection. The objective is to have a logic-based formal representation of this data collection, in order to offer researchers and data analysts a tool for accessing the ADNI data collection, and extracting subsets of data from it, in an intuitive manner. Furthermore, the *AD-Onto* also represents a data reference schema to be used by any other center involved in the gathering of data about Alzheimer’s disease, so contributing even to the harmonization of new data collections. The choice of ADNI is further justified at the end of the Background section.

In the rest of this section, we provide some insights about: (i) Alzheimer’s disease and the relevance of the neuropsychological tests for the assessment of the disease itself; (ii) an overview of the AD main data collections and AD Ontologies together with some recent literature on Biomedical Data Integration and Harmonization issues; (iii) the ADNI project, its data collection, and the motivations why we focused on it; (iv) a brief introduction to computational ontologies and methods for building them.

### The Alzheimer’s disease and the relevance of the neuropsychological assessment

Alzheimer’s disease (AD) is the most common form of dementia that develops gradually in the brain and slowly causes death. Any drug or treatment are not very effective and this has huge consequences for healthcare organizations and the economy around the world. According to the 2016 World Alzheimer’s Report [2] there are around 47 million people that live with dementia around the world and the estimation of the financial cost for addressing such diseases now is around 1 trillion dollars. The main symptoms of AD are the loss of memory and cognitive impairments, both deficits able to affect social and occupational activities [3]. Despite many attempts, so far no effective cure has been identified for AD and the average survival times from the onset of dementia is about 4.5 years, with a peak of 11 years for younger patients [4]. Early diagnosis and stratification of probable AD are crucial for the adoption of therapeutic strategies able to slow the progression of the disease.

In recent times, neuropsychological tests have become fundamental in the assessment of patients with Alzheimer’s disease and they are now used to provide confirmatory evidence for the diagnosis [5]; as we can read in the Diagnostic and Statistical Manual of Mental Disorders (DSM-5) [6], dementia is categorized as a neurocognitive disorder (NCD) and the primary clinic deficit is in the cognitive function. In 2011, the National Institute on Aging and the Alzheimer’s Association revised the clinical diagnostic criteria for Alzheimer’s disease [7] after 27 years: even if they updated the diagnosis guidelines, the core clinical criteria for dementia remain the evaluation of the cognitive and the behavioral (neuropsychiatric) abilities. Even decades before the actual diagnosis, these cognitive deficits are likely to be found in patients and they represent the preclinical phase of dementia [8, 9]. During this phase, patients present difficulties in remembering recent events, recalling conversations, and naming objects and persons, and families report events such as misplacement of items and repeating themselves [10]. Several studies, focused on this early stage, have found impairments mostly in episodic memory [9, 11], but also in executive functioning and perceptual speed [11].

The neuropsychological assessment includes several cognitive components, such as orientation, attention, naming, reading, recall information, writing, repeating, and copying: these abilities could be impaired in different stages of dementia. Testing these cognitive abilities is also important for evaluating changes in impairment, during and after treatment [5]: neuropsychological tests are sensitive measures and it’s, therefore, possible to detect the transition from preclinical AD to symptomatic AD [12]. For this reason, an early neuropsychological assessment is crucial to identify not only which cognitive domain is impaired, but also the degree of this impairment and which abilities are preserved, in order to make a correct diagnosis and to plan an efficient treatment, both pharmacological and neuropsychological.

### AD data collections and ontologies

In biomedical applications, data harmonization and integration are crucial tasks that must be performed to obtain data sets suitable to be analyzed by big data technologies, where traditional statistical and advanced ML techniques are mainly used. Many papers describe the role of ontologies in managing biomedical data [13–16]. In [17] a very broad review of data harmonization and integration methods is presented, and a new approach to address these problems is proposed. From these works it emerges that ontologies are fundamental tools for searching, harmonizing, and integrating data.

A lot of work has been done to build domain ontologies in many fields of the biomedical domain. Gene Ontology (GO), see [18, 19], is the first famous one, created to unify the world of genetics and still continuously updated with the contributions of all the discoveries of new sequences and new features. To have a fairly complete knowledge of the work of ontologies production it is useful to examine BioPortal (http://bioportal.bioontology.org), a comprehensive repository that hosts a large number of biomedical ontologies. BioPortal provides access via web services and web browsers to ontologies developed in OWL, RDF, OBO format and Protégé frames. However, it is evident that many of these ontologies have not been updated for years.

Literature is rich with articles on methods to create, manage and use ontologies, see [20] as an example of methods for data integration through ontologies, [21], a review of ontology data access methods, [15] that highlights ontologies’ potentialities to support integrative analysis and interpretation of multimodal data, [22] that illustrates the problem of ontology-mediated queries.

As far as Alzheimer’s disease is concerned a big effort was devoted to collecting data. Many projects around the world used huge amounts of resources to collect data in a standardized way. Most of these data collections are connected in the Global Alzheimer’s Association Interactive Network (GAAIN) [23, 24] (http://www.gaain.org/), the USA project that stores data from multiple places in a single location. GAAIN platform provides more than sixties datasets with half a million subjects, that can be explored through a user-friendly interface called *GAAIN Interrogator*.

Among EU initiatives, European Medical Information Framework (EMIF) and Dementias Platform UK (DP-UK) are among the richest in terms of the number of data sources and subjects. EMIF project (http://www.emif.eu/), was launched in 2013, with the purpose of improving the access of researchers to patient-level data from distinct health data repositories across Europe. A byproduct of this project is EMIF-AD (http://www.emif.eu/emif-ad-2/), containing a catalog of metadata of a multicenter study, which seeks to enable the finding, assessment, and use of preexisting data (see [25]). For querying the catalog, an interface has been proposed in [26] that allows using natural language, through an ontology, in order to support the data sets selection. It is noteworthy the following statement made in [27], where the authors analyze many studies on AD cohort data and try to extract a “data landscape”. They concluded that “a common semantic framework for patient-level AD data is needed to enable the community to work across cohort data sets and ultimately to generate robust scientific insights to advance AD research”.

On the other hand, as well as EMIF, also DP-UK [28] is a centralized resource designed to support researchers in the study of dementia and all its related disorders. The platform hosts a wide range of different types of data, including clinical and biological information, in addition to neuroimaging data, collected from multiple studies and research initiatives. Clinical data can be browsed through a unified entry point with a user-friendly interface, with the possibility to filter studies through a simple Microsoft Power BI interface according to a very limited set of criteria. Biological and neuroimaging data can be instead retrieved after the submission and the explicit approval of a formal request.

The data collection initiatives on Alzheimer’s disease vary greatly in the following parameters: the number of examined patients, the duration of follow-up, and the type of characteristics that are measured. These characteristics can be distinguished into demographic, clinical, genetic, biospecimen, morphological and functional brain features (brain images and EEG), and constitute the Alzheimer’s landscape. AD’s landscape is recently expanding to include new biomarkers and other aspects of differentiation such as environmental and ethnoracial ones. The following projects are examples where these aspects are addressed:

- the most recent works of Michael Weiner and his group presented in [29–31], where they propose to extend the enrollment of ADNI project to Black and Latin older adults, that show a higher risk of dementia, to investigate how health-care disparities and sociocultural factors influence potential AD therapies and prognosis greater risk for dementia.
- the Greek initiative HELIAD [32, 33] that investigates the aspect of diet and aging and their correlation with AD;
- the Argentine initiative AGA [34] aimed at identifying genes for susceptibility to sporadic AD and genes that modify phenotypes related to AD as TREM2, PLCG2, and ABI3 rare coding variants and to investigate various aspects of environmental factors.

About AD ontologies, they represent different landscapes of the disease depending on the purpose for which they are created. SWAN (Semantic Web Applications in Neuromedicine) [35] was one of the first focused on the storage and contextualization of the existent information about AD. SWAN is an ontology for modeling scientific discourses, and it was developed as a modular set of components. Then, a SWAN knowledge base is a database collecting knowledge about scientific discourse in one particular domain, as for instance the SWAN-ALZHEIMER knowledge base (https://www.w3.org/TR/hcls-swan/#swan-alzheimer), which is not available anymore.

In [36], an AD Ontology (ADO) is proposed, built using existing tools and extracting data from many sources: review articles, the content of online books, standard knowledge bases, encyclopedias, glossaries, and informative online sources, and websites. ADO has been designed to be useful for ontology-driven searching of the literature. In [37], the AD Map Ontology (ADMO) is proposed, in order to describe knowledge about AD-related biological pathways, based on systems biology terms. To conclude this short review on AD ontologies a recent proposal from a French-English collaboration of the Sorbonne university (Paris) and University College (London) must be mentioned. This research leads to generalizing the concept of process from biological processes (reductionist approach, see the case of beta amyloide) to qualitative processes of different types, like lifestyles. In [38] the concept of Disease-Relevant Process (DROP) is introduced, which becomes Disease-Associated Process (DAP) in [39], "a flexible concept that can unite different areas of study of AD from genetics to epidemiology to identify disease-modifying targets". This last study results in ADAPT ontology in [39], a tool that could help the AD community to ground debates around priority setting using objective criteria for the identifying of targets in AD". Unfortunately, the initiatives to build an ontology for AD have often failed to have great success, also due to the low interest of clinicians and researchers in the field. Many of them are no longer updated as shown in the review paper [40]. Furthermore, the purposes for which they were proposed were not to facilitate standardization, integration, or harmonization of data.

About federated and single project initiatives of data collection, even if they rely on sophisticated access interfaces, as in the case of DP-UK platform, they are not powered by any semantics-based facility to access data.

For this reason, we decided to build a computational ontology based on ADNI. Indeed, ADNI has a relevant number of features, patients and follow-ups and, according to Google Scholar, is one of the most cited data collection concerning Alzheimer’s disease^1^. In this way, the ADNI computational ontology becomes a useful tool for accessing the ADNI data collection and obtaining datasets that are good for Machine learning applications.

### The ADNI initiative and data collection

The Alzheimer Disease Neuroimaging Initiative (ADNI) (https://adni.loni.usc.edu/) was launched in the United States in 2004 as a public-private partnership, led by Principal Investigator Michael W. Weiner, MD. It is a multi-center project, with two aims:

- developing clinical, imaging, genetic, and biochemical biomarkers for an accurate and earlier diagnosis on a cohort of thousands of patients suffering from neurodegenerative diseases and highly characterized control subjects.
- testing whether serial magnetic resonance imaging (MRI), positron emission tomography (PET), other biological markers, and clinical and neuropsychological assessment can be combined to measure the progression of mild cognitive impairment (MCI) and early Alzheimer’s disease (AD).

Patients are followed with visits and periodic examinations. ADNI is focused on image diagnostics, which makes up the bulk of its database. In addition to neuroimaging, ADNI includes all the clinical data collected in the various visits to which the participants were, and still are, submitted over several years. The data have been systematized into thousands of variables of various kinds (numerical, binary, categorical, textual) that correspond to a very detailed protocol of neuropsychological tests, physiological and cardiological examinations, laboratory tests, and genotyping data. Moreover, ADNI provides also whole genome SNPs data, microarray gene expression profiles and DNA methylation profiles [41].

**Fig. 1:**
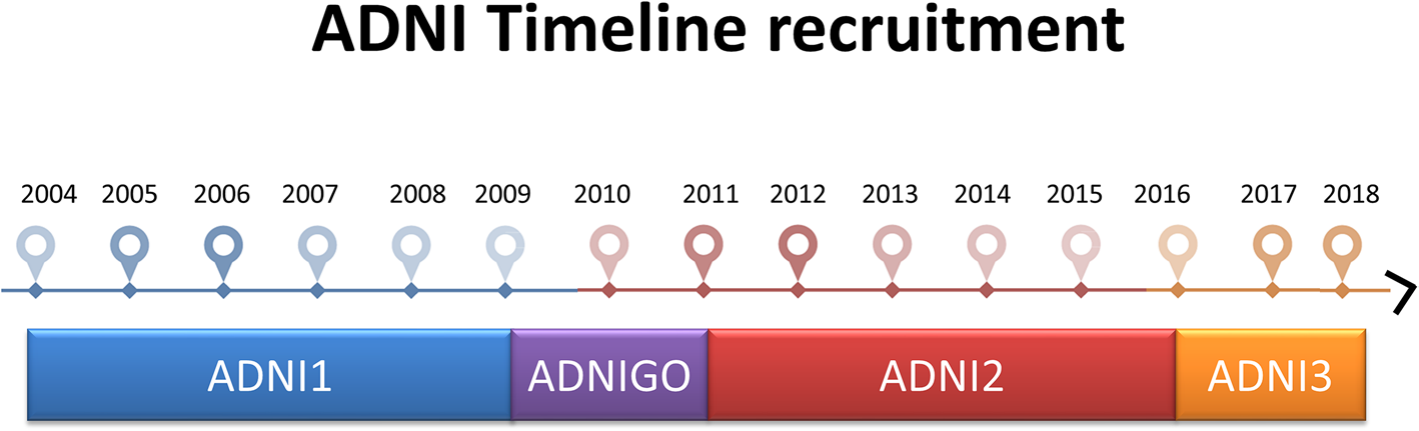
ADNI Timeline recruitment. Till now, the ADNI project has gone through four phases, namely ADNI1, ADNIGO, ADNI2 and ADNI3. During each phase new subjects have been enrolled and additional features have been observed

Over the years, ADNI has gone through several phases (Fig. 1), namely ADNI1 (2004-2009), ADNIGO (2009-2011), ADNI2 (2011-2016), and ADNI3 (started in 2016), each characterized by specific objectives. In particular, the ADNI1 phase was mainly oriented to biomarkers and MRI data. The study, financed by both public and private funds, gathered the clinical information of approximately 800 participants divided into AD, MCI (Mild Cognitive Impairment), and CN (Cognitive Normal). In 2009, while ADNI1 was still underway, a new phase of the study called ADNIGO started bringing 200 new participants all classified as MCI. The attention of this phase was oriented more on the measures of biomarkers in the early stages of the disease and on the improvement of brain measurements with MRI. ADNIGO lasted 2 years and in 2011 ADNI2 began, where new participants were added to the subjects still alive from the previous phases (diagnostic follow-up protocol). Only in this phase, two new classes of patients appeared: *early mild cognitive impairment* (EMCI) and *late mild cognitive impairment* (LMCI), which represent fine-grained levels of MCI. The main novelty at this phase was the introduction of new data types in the image category (e.g., amyloid PET). In 2016 ADNI2 gave way to ADNI3, still collecting data from old and new participants. For this phase, the study is emphasizing at most the importance of finding the correlations between heterogeneous data types such as clinical, genetic, imaging, and biomarkers data.

Till now, the ADNI project has enrolled about 2000 subjects. To date, the standard set up by ADNI still represents a well-established and widespread framework to deal with. In fact, ADNI is one of the biggest multi-center efforts in the world that collects data with shared protocols and, even if the number of participants to the study is not as large as those contained in some collections included in GAAIN [23], it is the one with the largest number of features that are measured. ADNI is worldwide acknowledged as one of the reference projects in the field of AD, for the quality, size, and accuracy of clinical data collected since its beginning. Indeed, it collects heterogeneous data from independent AD centers through very strict protocols that make these data standardized so that they can be easily analyzed altogether.

### Introduction to computational ontologies

Representational primitives are typically:

- classes, which represent concepts, e.g., MotorVehicle, Bicycle;
- attributes, e.g., maxSpeed, registrationDate, which are properties of a class that take values in basic types, e.g., Integer, Date;
- relations that are established between classes, as for instance hasInsurance that links the MotorVehicle and the InsuranceContract classes. A well-known relation is the is-a, which links a class to a more generic class, as in the case of MotorVehicle and TransportationMeans. The former assumes the role of sub-class, whereas the latter the role of super-class. Note that, a sub-class inherits all the attributes and relations of its super-classes. For instance, the MotorVehicle class inherits the maxSpeed attribute from the TransportationMeans class.

Attributes and relations are also referred to as properties.

An ontology also comprises individuals, which are classes’ instances, e.g., :myCar instanceOf MotorVehicle. We can also say that, the type of the :myCar individual is the MotorVehicle class. Individuals of a class can be described by assigning values and linking individuals to attributes and relations, respectively, in accordance with the defined properties. Definition of classes and properties identify the Terminology Box (TBox), whereas individuals represent the Assertion Box (ABox).

Computational ontologies are used for many purposes, which go from the definition of a common understanding shared among people, up to the representation of background knowledge for logic-based reasoning systems. Furthermore, depending on the purpose, computational ontologies may have different shapes. For instance, when it is used for classification purposes, it can be limited to a taxonomy (i.e., a hierarchy of classes and sub-classes), whereas when it is used for data integration and interoperability, a fully structured ontology with attributes definition is required.

In order to be machine-readable, an ontology must be specified in a formal language. Currently, the *de-facto* standard language for representing computational ontologies is the Ontology Web Language (OWL) [42], which is rooted in the Description Language formalism. OWL is part of the Semantic Web Stack^2^, a layered architecture based on XML and RDF (Resource Description Framework) that shows how technologies that are standardized for the Semantic Web are organized to make the Semantic Web possible. OWL ontologies can be inspected by means of SPARQL [43], an SQL-like language for posing queries on RDF documents.

Many approaches for building ontologies exist, and they are often classified according to the type of input used for learning: unstructured data, semi-structured data, and structured data. Unstructured data are represented by textual documents, which can be web pages, social media posts, emails, and technical reports, and for learning ontologies from them, natural language techniques are mainly applied. Semi-structured data are characterized by the lack of a rigid and formal structure, and they usually contain tags for separating semantically relevant knowledge from textual content. In the case of semi-structured data, learning ontologies mostly requires data mining and web content mining techniques. See [44] and [45] for an extensive survey on ontology-building tools from textual documents and semi-structured data, respectively. Structured data are those kinds of data that are created by using a predefined and fixed schema, typically organized in a database. Also, in this case, many approaches exist and they mainly consider relational databases as input, where meta information like primary and foreign keys are exploited to build relationships between learned concepts. See [46] for an overview of ontology learning methods from relational databases.

From what we reported in the background, we can derive the following motivations for choosing the ADNI data model as a reference for our ontology:

- our exam about types of information contained in a large number of data collection and ontologies showed that the ADNI data model contains most of the attributes of these data sets and is one of the largest in terms of the number of different types of gathered data;
- other initiatives, like GAAIN and EMIF, are federated models that would require a common ontology to align data from heterogeneous sources. Both GAAIN and EMIF contain ADNI, which could become their reference ontology;
- other research projects for collecting and analyzing data, as for instance AddNeuroMed [47]) and some centers for the diagnosis and treatment of neuro-diseases choose to use subsets of ADNI data set (see the AD research group of the Department of Medicine and Surgery, University of Perugia, Italy);
- an immediate byproduct of an ontology that organizes the knowledge contained in ADNI could be to allow researchers to fully take advantage of this big and rich data repository, through easier applications for querying the provided data;
- finally, the ADNI data collection is definitely the most utilized source for those analyzing data on Alzheimer’s disease, as evidenced by the number of citations received.

Finally we set out the objectives of our work:

1. to define a conceptualization of the ADNI data collection and a first kernel of the computational ontology focusing on the domain of the neuropsychological tests;
2. to illustrate the design and implementation of a software tool for populating the developed ontology with the ADNI data;
3. to show the benefits of such a solution through the description of two currently implemented use cases and the other possible use cases that will be developed in future work.

## Methods

As anticipated, the objective of this work is to build a computational ontology from the ADNI data collection^3^. The ADNI data collection is composed of a set of *comma separated value* (CSV) files, which have a tabular form. They mainly contain numeric values and then, and they do not require any natural language processing. However, they can not be treated as a relational database, since meta information, such as integrity constraints, are not defined.

In order to build the TBox of the *AD-Onto*, we decided first to provide a conceptualization of the data collection and then to use such a conceptualization as a reference for realizing the OWL implementation. In the next paragraph, the performed conceptualization is presented. Concerning the ABox, a set of mapping rules between columns of the ADNI files and *ontology paths* were defined. Then, these rules were used for implementing the desired data import modules. The definition of mapping rules is outlined in the next paragraph.

### Conceptualization of the ADNI data collection

“A body of formally represented knowledge is based on a conceptualization: the objects, concepts, and other entities that are assumed to exist in some area of interest and the relationships that hold among them. A conceptualization is an abstract, simplified view of the world that we wish to represent for some purpose. Every knowledge base, knowledge-based system, or knowledge-level agent is committed to some conceptualization, explicitly or implicitly”.

The quote above, originally appeared in [48], was also referred by Gruber in his seminal paper on computational ontologies [49]. Hence, the objective of a conceptualization is the identification and modeling of the entities and relationships between them to provide a description of a given application domain, with respect to a defined purpose. In our case, the objective was to build a computational ontology suitable for representing data about Alzheimer’s disease, and of the ADNI collection in particular. In order to perform such an activity, we analyzed the documents available in the ADNI web portal, as well as the files containing the actual data. In the following paragraph, we started addressing an overall conceptualization of the whole ADNI data collection, and we subsequently focused on the neuropsychological domain. In the Supplementary Material, we then focus on further subdomains, for which we illustrate the conceptualization.

#### An overall conceptual model of the ADNI data collection

In this step, we focused on a high-level view of the ADNI data collection, with the objective to provide an overall conceptual model of its content.

**Fig. 2:**
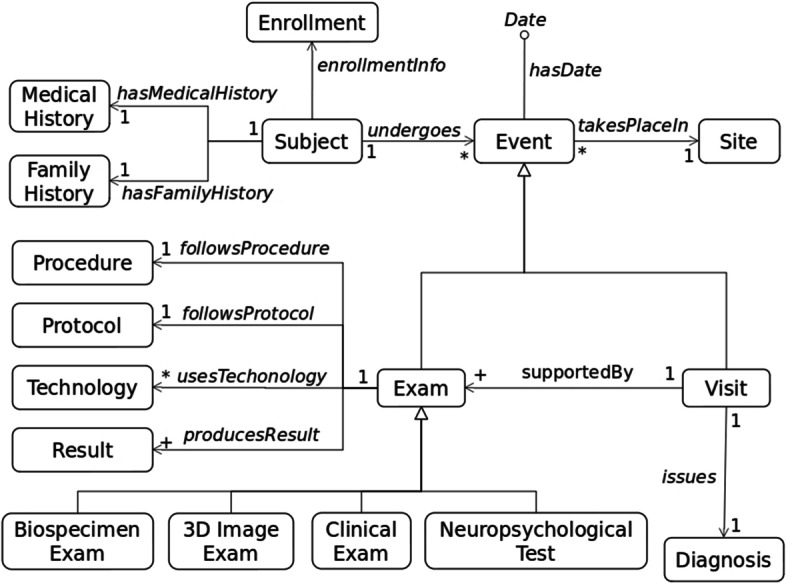
An overall conceptual model of the ADNI data collection. The main classes and relations between classes representing an overview of the entities in the ADNI database

Figure 2 represents a UML class diagram showing the high-level classes and relationships that reflect the content of the whole ontology. Subject represents the class of the people that are under observation in the project, e.g., ADNI. MedicalHistory and FamilyHistory represent the classes reporting health information (e.g., diseases, drugs) related to findings issued and decisions taken outside the project and about a Subject, and her/his relatives, respectively. Enrollment is the class concerning the adherence to the criteria for becoming a subject of the project. Event represents the class of the episodes that a Subject undergoes in the framework of the project^4^.

An Event is characterized by a date (hasDate attribute), which reports when the event occurred and is extremely useful to perform longitudinal studies. An Event takesPlaceIn a Site, which represents the class of the medical centers participating in the project. An Event can be either a Visit or an Exam. The Visit class represents the scheduled appointments during which a Diagnosis is issued on the basis of (supportedBy) some exams. An Exam can be of four different types: BiospecimenExam, which is the class of the exams that are performed on biological materials, e.g., urine, blood, DNA; 3D ImageExam, which is the class of the exams that capture images of the activity of the brain in order to see how the brain is functioning, i.e., Positron Emission Tomography (PET) and Magnetic Resonance (MR); ClinicalExam, which represents the class of examinations such as ECG, and vital signs checking; NeuropsychologicalTest, which represents the class of the neuropsychological tests. Each Exam follows a Procedure and a Protocol, can use some technologies (Technology class), and produces some results (Result class).

#### Neuropsychological test conceptualization

**Fig. 3:**
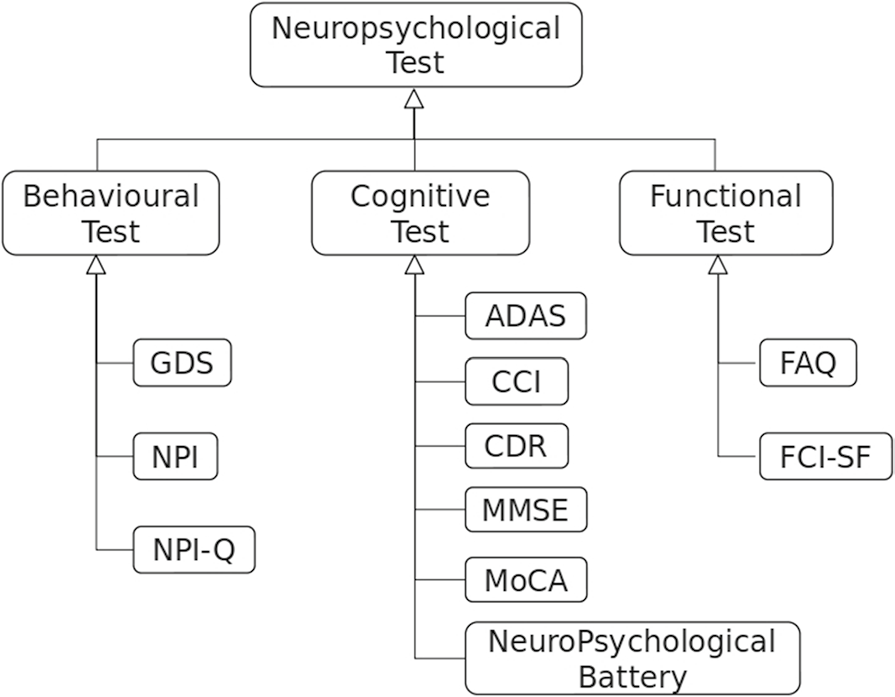
Neuropsychological tests taxonomy. Neuropsychological tests, which are the leaves of the taxonomical tree, are partitioned into behavioural, cognitive, and functional tests

The NeuropsychologicalTest class is the most general class that represents any test performed through an interview in order to evaluate the neuropsychological status of a subject. Neuropsychological tests can screen for cognitive, behavioral, and functional abilities [50]. According to that, as shown in Fig. 3, the generic NeuropsychologicalTest class has been specialized into three classes, namely, BehaviouralTest, CognitiveTest, and FunctionalTest. The leaves of this taxonomy represent the specific typologies of tests that are performed within the ADNI project. The GDS class (Geriatric Depression Scale), the NPI class (Neuropsychiatric Inventory), and the NPI-Q class (Neuropsychiatric Inventory Questionnaire) have been modelled as sub-classes of the BehaviouralTest class. The ADAS class (Alzheimer’s Disease Assessment Scale), the CCI class (Cognitive Change Index), the CDR class (Clinical Dementia Rating), the MMSE class (MiniMental State Exam), the MoCA class (Montreal Cognitive Assessment), and the Neuropsychological Battery class have been modelled as sub-classes of the CognitiveTest class. The FAQ class (Functional Activity Questionnaire), and the FCI-SF class (Financial Capacity Instrument - Short Form), have been modeled as sub-classes of the FunctionalTest class.

*Neuropsychological test items.* Each neuropsychological test is organized according to a set of questions, here referred to as neuropsychological items, and represented by the NeuropsychologicalItem class. Each item aims at evaluating a specific aspect (or scope) of the psychological status of a subject. For instance, the Mini-Mental State Exam (MMSE) is composed of 30 questions organized as follows: 10 questions are evaluating orientation capabilities with respect to both space and time; 3 questions are for evaluating registration capabilities, i.e., to record information; 5 questions are about attention and calculation capabilities, e.g., to count or spell a word backward; 3 are about recall capabilities, and 9 about language and praxis. For instance, in order to check recall capabilities the following questions are posed: “Earlier I told you the names of three things. Can you tell me what those were?”. The answers to this question have been modeled with the following three classes: MMSE_RecallItem_1, MMSE_RecallItem_2, and MMSE_RecallItem_3. We classified all the items according to two criteria. The first criterion concerns the scope of the item, i.e., the specific aspect it aims at evaluating. We defined a set of classes representing the psychological sub-domains addressed by the items, and we classified each item with respect to the sub-domain it refers to. For instance, the MemoryItem class represents the generic class of the items concerning memorization and recall capabilities, and all the items concerning memory issues, e.g., MMSE_RecallItem_1 have been defined as subclasses of the MemoryItem class. In particular, the scopes modeled in the ontology are the following:

- DailyFunctioning, which is specialized into ActivitiesOfDailyLiving, HomeAndHobbies, and PersonalCare;
- ExecutiveFunction, which is specialized into CognitiveFlexibility, in addition to JudgmentAndProblemSolving, and ProcessionSpeed;
- Language, which is specialized into Reading, VerbalFluency, Comprehension, Naming, Repetition, and Writing;
- Memory, which is specialized into Learning, LogicalMemory, Long-TermMemory, Registration, SemanticMemory, Short-TermMemory, WorkingMemory, and MemoryLoss;
- Orientation, which is specialized into PlaceOrientation and TimeOrientation;
- PsychiatricDomain, which is specialized into Disinhibition, Anxiety, Apathy/Indifference, Depression, Dysphoria, Agitation/Aggression, EatingDisorders, Elation/Euphoria, Hallucinations, SleepDisorders, Delusion, and Irritability/Lability;
- SocialFunctioning, which is specialized into CommunityAffair;
- Visuo-SpatialAbility, which is specialized into VisualConstruction.

The second criterion concerns the type of test each item belongs to. Indeed, all the items of each neuropsychological test, have been defined as sub-classes of a more generic class. For instance, the MMSEItem class represents the generic class of all the items of the MMSE test.

**Fig. 4:**
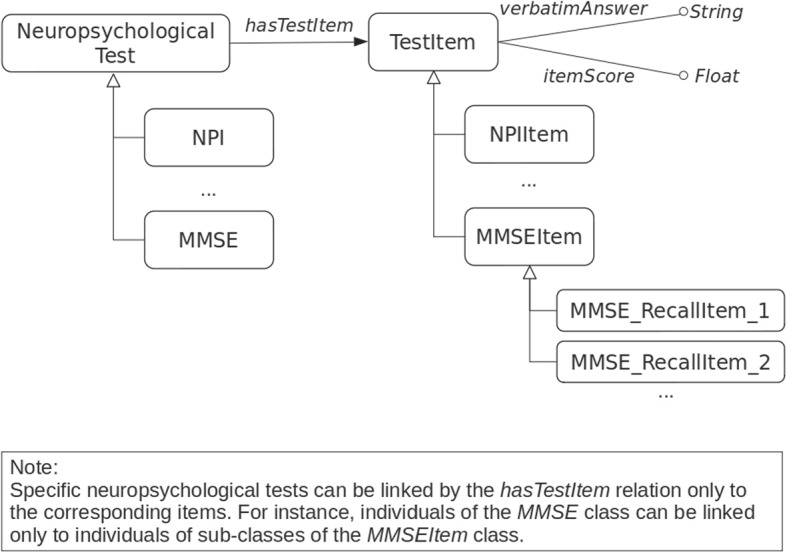
Linking neuropsychological tests and items. Each neuropsychological test has a set of items representing the question posed to the subject during the interview. Each item can be associated with the answer given by the subject and the score given by the medical doctor

*Relations and Attributes.* In order to associate an item to a neuropsychological test, the hasTestItem association has been defined between both the NeuropsychologicalTest and TestItem classes. However, this is a generic association that does not consider specificity of tests and items. In fact, it is expected that each test can be linked only to questions it is composed of. For instance, it is expected that individuals of the MMSE class are linked only to individuals of the MMSEItem class. In the conceptualization, we did not model such a constraint^5^. However, in Fig. 4 a text note describes this constraint to be taken into account in the ontology implementation. In order to represent the actual values of the ADNI data collection, class attributes have been defined. For instance, the TestItem class, has the verbatimAnswer and the itemScore attributes, as shown in Fig. 4. The former, whose type is *String*, allows the answer given to a question to be represented, and the latter, whose type is *Float*, the score associated with it.

### Rules for mapping the ADNI data collection and its conceptual model

In order to represent the actual ADNI data in accordance with the *AD-Onto* representation, we defined a set of mapping rules between the structure of the ADNI data collection and the conceptual model previously introduced. Hence, such mapping rules have been used as technical specifications for the implementation of the import data module that will be illustrated in the Results section.

**Fig. 5:**
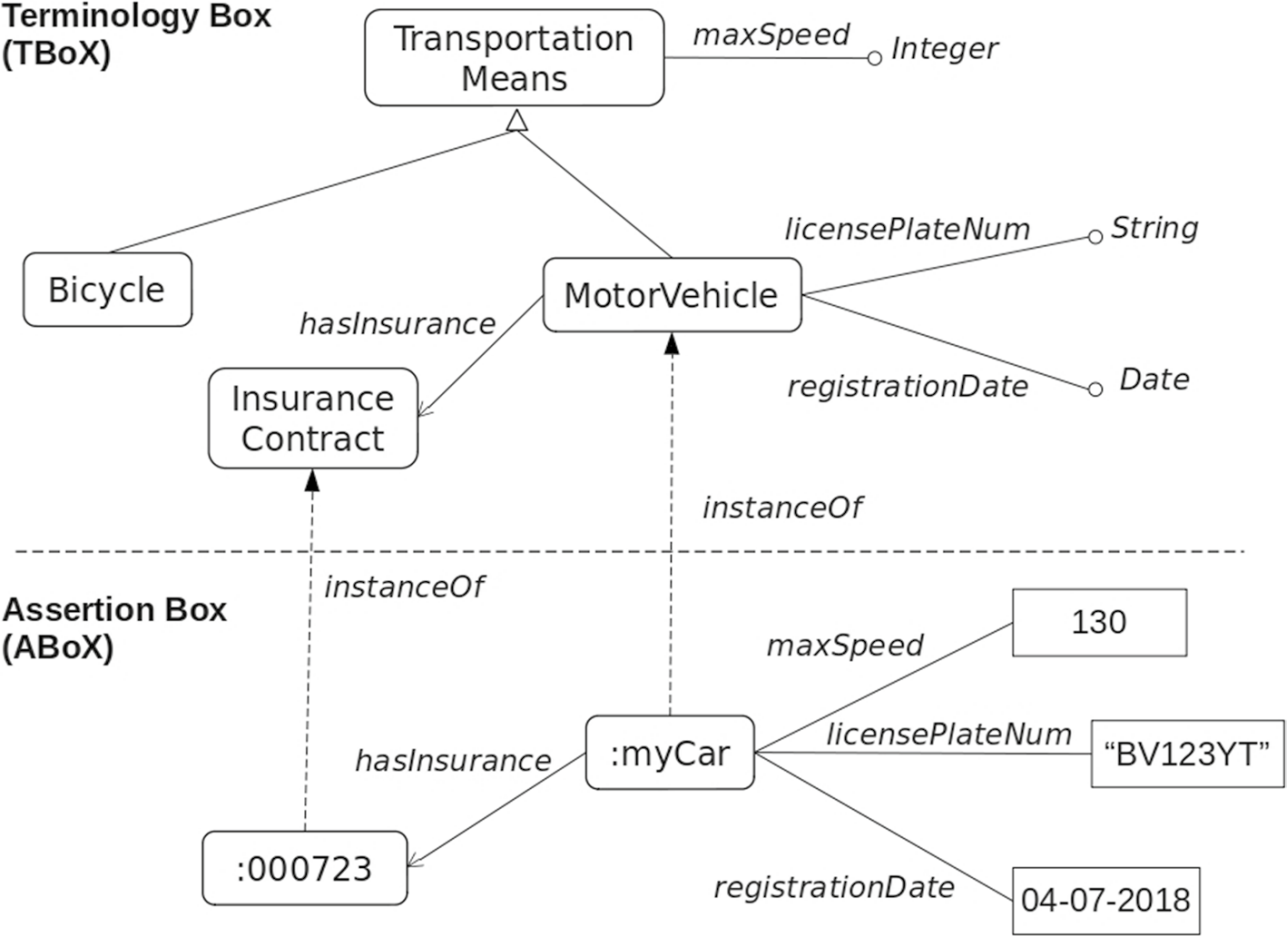
An example of conceptual model in the *Transport* domain. Knowledge is organized in a conceptual level (TBox), where classes and properties are defined, and an extensional level (ABox), where instances, i.e, individuals of the real world are introduced

For defining mapping rules, we first distinguish between paths at TBox level and paths at ABox level. The former are paths in the graph representing the conceptual model that start from a class and end in a base type through a sequence of properties and classes. The latter are paths starting from an instance of a class and ending in a valued attribute. An ABox path can be considered as an instance of a TBox path, i.e., classes and base types are substituted with individuals and actual values. Paths are here represented by using a dot notation, i.e., paths’ elements are separated by a dot. For instance, considering Fig. 5, the path MotorVehicle.licensePlateNum.String is a TBox path, whereas the path :myCar.licensePlateNum."BV123YT" is an ABox path.

The ADNI data collection is divided into a set of files, all having a tabular form. Each column of each table must be mapped with a TBox path. For instance, the column *MMDATE* of the *MMSE* table represents the score assigned to the answer given to the “What is today’s date?” question while carrying out the MMSE test. According to that, the *MMDATE* column corresponds to the following path at TBox level: MMSE.hasTestItem.MMSE_OrientationItem_1.0_1_score.Float. This represents the TBox path that links the MMSE class to the score associated with the answer to the above question. Furthermore, according to the conceptual model in Fig. 2, a subject undergoes an event, and then she/he undergoes an MMSE test. Consequently, in order to connect the Subject class to the above score, the whole path at TBox level is Subject.undergoes.MMSE.hasTestItem.MMSE_OrientationItem_1.0_1_score.Float.

Here, we define a *mapping rule* for a field of a table as a correspondence between the field and a TBox path, and we use the symbol "->" between the former and the latter. For instance, the mapping rule for the field MMDATE of the *MMSE* table is shown in Listing 1:

**Figure:**
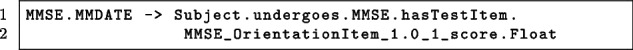
**Listing 1** The mapping rule for the *MMDATE* field of the *MMSE* ADNI table

Mapping rules will be applied in order to generate ABox paths and populate the ABox with data from the ADNI tables. For instance, a row in the *MMSE* table reports all the data collected during a single *MMSE* session. In particular, the *RID* column reports the identifier of the subject performing the test, and the *MMDATE* column the score assigned to the “What is today’s date” question. If we select a row with *RID* equals to 134 and *MMDATE* equals to 1, the corresponding ABox path will be the following: Subject:134.undergoes.MMSE:1.hasTestItem.MMSE_OrientationItem_1:1.0_1_score.1, where Subject:134 represents the individual of the Subject class corresponding to the subject with *RID* equals to 134, MMSE:1 is the instance of the MMSE class collecting the information about the session of the *MMSE* test corresponding to the considered row in the *MMSE* table, and MMSE_OrtientationItem_1:1 is the instance of the MMSE_OrtientationItem_1 that refers to the “What is today’s date” question of that *MMSE* session.

In defining the mapping rules, we need to cope with different implementation choices adopted during different phases of the ADNI project. In fact, there are cases in which the same piece of information is represented by different fields depending on during which ADNI phase it was acquired. For instance, during the ADNI1 phase, the date in which an MMSE test is performed was represented by means of the EXAMDATE field, whereas, during the following ADNI phases by means of the USERDATE field. However, since both the fields have the same semantics, they correspond to the same TBox path, i.e., Subject.undergoes.MMSE.hasDate.Date, and originate the two mapping rules shown in Listing 2:

**Figure:**
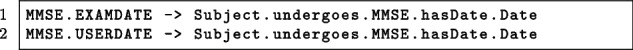
**Listing 2** An example of mapping rules with the same right end

## Results

This section aims at illustrating the concrete outcomes of the performed activities, i.e., the development of the ontology, and the software tool for populating the ontology with the ADNI from ADNI. In particular, some fragments of the OWL code implementing the ontology, and the macro-architecture of the *OntoLoader* tool are illustrated.

### Implementation of the *AD-Onto*

The conceptualization outlined in the previous paragraph is used as a reference for implementing the actual ontology, the *AD-Onto*. The implementation language is OWL, which is the *de facto* standard for representing computational ontologies. OWL is a logic-based language and is part of the W3C’s Semantic Web technology stack [51]. Knowledge expressed in OWL can be reasoned with by computer programs either to verify the consistency of that knowledge or to make implicit knowledge explicit.

The fragment of the *AD-Onto* shown in Listing 3 is focused on the *MMSE* neuropsychological test. The first line states that MMSE is a class. In particular, http://www.modiag.it#MMSE is the Uniform Resource Identifier (URI) of the class that is being defined. The second and third lines state that the MMSE class can be named either as *Mini-Mental State Examination* or as *MMSE*. Assigning more labels allows the definition of synonyms. The fourth line states that the MMSE class is a subclass of the CognitiveTest class, which is defined elsewhere in the *AD-Onto*. In fact, the rdfs:subClassOf implements the *is_a* relationship, which has been introduced in the Background section. The rest of the fragment implements the constraint defined in the text note in Fig. 4, which in the case of the MMSE test states that the property hasTextItem can be valued only with individuals of the MMSEItem resource, which is another class defined elsewhere in the ontology.

**Figure:**
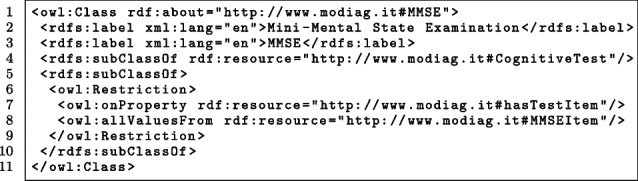
**Listing 3** OWL fragment about the deinition of the *MMSE* class

A relevant aspect of the performed implementation is the involvement of existing ontologies that address specific topics dealing with Alzheimer’s disease. For instance, the NCI Thesaurus OBO Edition [52] is a reference terminology that includes broad coverage of the cancer domain, including cancer-related diseases, findings, and abnormalities. In particular, it defines a lot of resources addressing neuropsychological tests, e.g., the resource http://purl.obolibrary.org/obo/NCIT_C74982 defines the MMSE test. For instance, in the *AD-Onto*, the fragment shown in Listing 4 is present:

**Figure:**
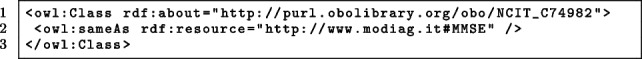
**Listing 4** An example of link between the *AD-Onto* and an existing ontology

which states that the NCIT_C74982 and the MMSE classes refer to the same thing. In fact, the owl:sameAs statements are often used in defining mappings between ontologies, since it is unrealistic to assume everybody will use the same name to refer to individuals. In this way, resources defined in the *AD-Onto* ontology are mapped to resources defined in existing ontologies and this represents a further step towards interoperability. In fact, for instance, whenever a given resource is categorized or annotated as a NCIT_C74982 from the *OBO* thesaurus, we can infer that it is also annotated with the MMSE class defined in the *AD-Onto*. Additional ontologies that have been integrated into the *AD-Onto* are: the Drug Ontology (https://obofoundry.org/ontology/dron.html), the International Classification of Diseases (https://www.who.int/standards/classifications/classification-of-diseases), and the Symptom Ontology (https://obofoundry.org/ontology/symp.html) for specifying drugs, diseases and symptoms in the context of the medical history.

### The data import software component

**Fig. 6:**
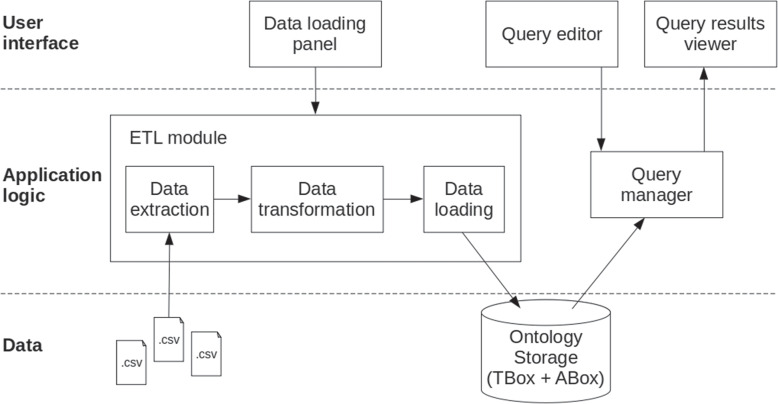
Macro-architecture of the *OntoLoader* software component. The *OntoLoader* is organized according to a three layer architecture: user interface, application logic, and data layer

Figure 6 depicts the macro-architecture of the *OntoLoader*, the software component devoted to the import of data into the ontology. In addition, the component allows functionalities for querying the ontology. Three layers can be distinguished: (i) the user interface, (ii) the application logic, and (iii) the data.

The user interface provides access to the functionalities for: (i) importing data through the *Data import panel*, by selecting the data to be imported and triggering the import task; (ii) querying data through the *Query editor*, and the *Query results viewer*. In particular, the former allows a SPARQL [43] query to be written, whereas the latter allows query results to be shown.

The application logic performs the actual import task in accordance with the Extraction, Transformation, and Load (ETL) approach. In particular, the *Data extraction* module is in charge of acquiring the data from the source, which in the case of the ADNI data collection is a *comma separated value* (*.csv*) file. The *Data transformation* module applies the rules previously described, in order to make the data compliant with the *AD-Onto*. The *Data loading* module stores the transformed data in the ontology, as part of the ABox. Furthermore, the *Query manager* performs the query on the ontology and retrieves the requested data to the user interface.

The *OntoLoader* component has been developed as a Java application and the ontology storage is ensured by the TDB triple store, which is a component of the Apache Jena library [53]. It allows importing data about neuropsychological tests from the ADNI data collection.

## Discussion

In this section, we discuss possible applications (use cases) of our tool.

Two of them have been already implemented:

- Application 1: Population of the ontology performed by the *OntoLoader* tool, which has been described in Results section.
- Application 2: Semantic querying of the data shown in the rest part of this section.

Concerning semantic querying, thanks to the formal representation of the ontology, data can be intuitively queried and reasoned with. For instance, the query in Listing 5 searches for items of neuropsychological tests that pertain to topics related to memory. In particular, it returns the identifiers of the subjects (?subject), the date when the test was performed (?date), the type of the item (?i_type) and the score (?score) obtained by the subject for that item.

**Figure:**
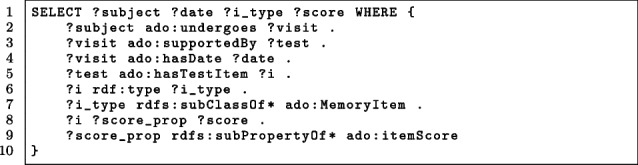
**Listing 5** SPARQL query for extracting memory-related data

In particular, lines 5-7 of the query constrain the variable ?i, which represents the ado:TestItem, to be an instance of the ado:MemoryItem class that represents the memory topic. This implies that the returned items, thanks to the semantics of the rdfs:subClassOf predicate, i.e., of the is_a relationship, are both those explicitly defined as instances of the ado:MemoryItem class, and those that are instances of sub classes of the ado:MemoryItem class, as for example the ado:Registration and ado:Learning classes.

Differently, consider the case in which the data are stored in *.csv* files, for instance in the ADNI data collection. In order to extract the same data as the query above, it is needed to address every single file dealing with neuropsychological tests and select only the columns related to the memory topic. This step implies knowing the meaning of columns in each file, which usually are named by non-intuitive labels, and then resulting in an error-prone activity.

Similarly, this happens in the case data are stored in traditional database management systems, such as for instance relational databases. In fact, even if in this case data extraction is supported by the SQL language, it is assumed that the structure of each table is known. In addition, relational databases do not natively support the is_a relationship and inheritance.

This way of performing queries allows data interoperability for multidimensional data analysis.

In the rest of this section, we introduce some possible extensions and additional applications of *AD-Onto* that are its scalability and its potential as a tool to support the integration and harmonization of data from different sources. Scalability, in the sense of enriching the conceptual model by adding new concepts, can be achieved by adding new classes and attributes with simple operations. Performing integration and harmonization of data from different sources requires mapping the variables of the new sources to the classes and properties in *AD-Onto*. To perform this mapping, we will investigate also the possibility of using ontology embeddings to annotate data sets, which appears to be very promising. The development of APIs for performing this task will be the subject of future research work.

## Conclusions

In this work, the main activities and results concerning the building of a computational ontology, namely the *AD-Onto*, for representing Alzheimer’s disease data have been outlined. The ontology is inspired by the ADNI data collection and was built by realizing a conceptual model, used as a specification for the actual OWL implementation. A software component, namely *OntoLoader*, has been illustrated. *OntoLoader* is in charge of automatically loading the ADNI data, currently only those related to the neuropsychological tests, into the ontology, and providing query functionalities on those data.

As illustrated in the Discussion section, organizing data according to an ontology gives the chance to query them in a more intuitive manner. This can be particularly helpful when data need to be extracted for being analyzed for applying, for instance, machine learning techniques. In fact, in these cases, even if it is expected that data analysts are aware of the meaning of the data, they should not be involved in technical details concerning their representation.

### Supplementary Information

**Additional file 1.**

## Data Availability

Data used in the preparation of this article were obtained from the Alzheimer’s Disease Neuroimaging Initiative (ADNI) database (https://adni.loni.usc.edu) and cannot be publicly provided because of their data use policy (https://adni.loni.usc.edu/wp-content/uploads/how_to_apply/ADNI_DSP_Policy.pdf). The *AD-Onto* ontology and the *OntoLoader* Java tool for importing ADNI data into the ontology can be downloaded at https://drive.google.com/drive/u/1/folders/1ZJtyyBqwjRXW9ZFi-W3EyvxxvguMhacZ.

## Abbreviations

AD: Alzheimer’s Disease
ADNI: Alzheimer’s Disease Neuroimaging Initiative
EMCI: Early Mild Cognitive Impairment
EMIF: European Medical Information Framework
ETL: Extraction, Transformation, and Load
GAAIN: Global Alzheimer’s Association Interactive Network
GO: Gene Ontology
LMCI: Late Mild Cognitive Impairment
MCI: Mild Cognitive Impairment
ML: Machine Learning
MMSE: Mini-Mental State Exam
MRI: Magnetic Resonance Imaging
NCD: Neurocognitive Disorder
OBDA: Ontology-Based Data Access
OBO: Open Biomedical Ontologies
PET: Positron Emission Tomography
PPMI: Parkinson’s Progression Markers Initiative
RDF: Resource Description Framework
SPARQL: SPARQL Protocol and RDF Query Language
SQL: Structured Query Language
OWL: Web Ontology Language
UML: Unified Modeling Language
XML: eXtensible Markup Language

## Footnotes

1 Searching on Google Scholar with "Alzheimer’s Disease Neuroimaging Initiative" the number of retrieved citations is about 100K.

2 https://www.w3.org/Consortium/techstack-desc.html

3 Data used in the preparation of this article were obtained from the Alzheimer’s Disease Neuroimaging Initiative (ADNI) database (https://adni.loni.usc.edu).

4 According to the UML notation, an arrow representing an association can be enriched with a label and cardinality constraints. For instance, the cardinality constraints attached to the undergoes relation says that one subject can be linked to zero or more events

5 Note that, in UML more sophisticated constraints can be modeled by using the Object Constraint Language (OCL)
