# Supplementary material for: An ontology-based approach for modelling and querying Alzheimer’s disease data
Source: BMC Med Inform Decis Mak. 2023 Aug 8;23:153. doi: 10.1186/s12911-023-02211-6 (PMC10408169; doi:10.1186/s12911-023-02211-6)
Supplement: Supplementary file 1 — Additional file 1. [file 12911_2023_2211_MOESM1_ESM.zip › 12911_2023_2211_MOESM1_ESM.pdf]

¡

RESEARCH

Supplementary Material for An Ontology-based approach for Modelling and Querying Alzheimer's Disease Data

Francesco Taglino[1*], Fabio Cumbo[1,2], Giulia Antognoli[1], Ivan Arisi[3], Mara D'Onofrio[3], Federico Perazzoni[4], Roger Voyat[5], Giulia Fiscon[6], Federica Conte[1], Marco Canevelli[7], Giuseppe Bruno[7], Patrizia Mecocci[8,9], Paola Bertolazzi[1] and for the Alzheimer's Disease Neuroimaging Initiative [†]

*Correspondence: francesco.taglino@iasi.cnr.it
[1] Institute of Systems Analysis and Computer Science "Antonio Ruberti" (IASI), National Research Council (CNR), Via dei Taurini 19, 00185, Rome, Italy
Full list of author information is available at the end of the article
[†]Data used in preparation of this article were obtained from the Alzheimer's Disease Neuroimaging Initiative (ADNI) database (https://adni.loni.usc.edu). As such, the investigators within the ADNI contributed to the design and implementation of ADNI and/or provided data but did not participate in analysis or writing of this report.

Further conceptualization results

In this supplementary material, further conceptualization results about the *AD-Onto* are shown. In particular, the following classes are outlined: *Family History* and *Medical History* (see Figure 7), *Biospecimen Exam* (see Figure 8), *Genetic Exam* (see Figure 9), and *3D Image Exam* (see Figure 10).

Author details
[1] Institute of Systems Analysis and Computer Science "Antonio Ruberti" (IASI), National Research Council (CNR), Via dei Taurini 19, 00185, Rome, Italy. [2] Genomic Medicine Institute, Lerner Research Institute, Cleveland Clinic, 9500 Euclid Avenue, 44195, Cleveland, Ohio, USA. [3] European Brain Research Institute (EBRI) "Rita Levi-Montalcini", Viale Regina Elena 295, 00161, Rome, Italy. [4] Department of Engineering, Uninettuno International University, Corso Vittorio Emanuele II 39, 00186, Rome, Italy. [5] Department of Engineering, University of Roma Tre, Via della Vasca Navale 79/81, 00146, Rome, Italy. [6] Department of Computer, Control, and Management Engineering "Antonio Ruberti", Sapienza University of Rome, Via Ariosto 25, 00185, Rome, Italy. [7] Department of Human Neuroscience, Sapienza University of Rome, Via Ariosto 25, 00185, Rome, Italy. [8] Department of Medicine and Surgery, University of Perugia, Piazzale Gambuli 1, 06129, Perugia, Italy. [9] Division of Clinical Geriatrics, NVS Department, Karolinska Institutet, Nobels väg 5, Solna, 17177, Stockholm, Sweden.

References

1. Voosen P. How AI detectives are cracking open the black box of deep learning. Science. 2017;.
2. Prince MJ, Comas-Herrera A, Knapp M, Guerchet MM, Karagiannidou M. World Alzheimer Report 2016-Improving healthcare for people living with dementia: Coverage, quality and costs now and in the future. Alzheimer's Disease International; 2016.
3. Bird T. Alzheimer's disease and other primary dementias Harrison's Principles of Internal Medicine ed E Braunwald et al. New York: The McGraw-Hill Companies Inc; 2001.
4. Xie J, Brayne C, Matthews FE. Survival times in people with dementia: analysis from population based cohort study with 14 year follow-up. bmj. 2008;336(7638):258–262.
5. McKhann G, Drachman D, Folstein M, Katzman R, Price D, Stadlan EM. Clinical diagnosis of Alzheimer's disease: Report of the NINCDS-ADRDA Work Group* under the auspices of Department of Health and Human Services Task Force on Alzheimer's Disease. Neurology. 1984;34(7):939–939.
6. Edition F, et al. Diagnostic and statistical manual of mental disorders. Am Psychiatric Assoc. 2013;21(21):591–643.
7. McKhann GM, Knopman DS, Chertkow H, Hyman BT, Jack Jr CR, Kawas CH, et al. The diagnosis of dementia due to Alzheimer's disease: Recommendations from the National Institute on Aging-Alzheimer's Association workgroups on diagnostic guidelines for Alzheimer's disease. Alzheimer's & dementia. 2011;7(3):263–269.
8. Payton NM, Rizzuto D, Fratiglioni L, Kivipelto M, Bäckman L, Laukka EJ. Combining cognitive markers to identify individuals at increased dementia risk: influence of modifying factors and time to diagnosis. Journal of the International Neuropsychological Society. 2020;26(8):785–797.
9. Mortamais M, Ash JA, Harrison J, Kaye J, Kramer J, Randolph C, et al. Detecting cognitive changes in preclinical Alzheimer's disease: A review of its feasibility. Alzheimer's & dementia. 2017;13(4):468–492.
10. Baldwin S, Farias ST. Neuropsychological assessment in the diagnosis of Alzheimer's disease. Current protocols in neuroscience. 2009;49(1):10–3.
11. Bäckman L, Jones S, Berger AK, Laukka EJ, Small BJ. Cognitive impairment in preclinical Alzheimer's disease: a meta-analysis. Neuropsychology. 2005;19(4):520.
12. Schindler SE, Jasielec MS, Weng H, Hassenstab JJ, Grober E, McCue LM, et al. Neuropsychological measures that detect early impairment and decline in preclinical Alzheimer disease. Neurobiology of aging. 2017;56:25–32.

13. Bodenreider O. Biomedical ontologies in action: role in knowledge management, data integration and decision support. Yearbook of medical informatics. 2008;17(01):67–79.
14. Rubin DL, Shah NH, Noy NF. Biomedical ontologies: a functional perspective. Briefings in bioinformatics. 2008;9(1):75–90.
15. Hoehndorf R, Schofield PN, Gkoutos GV. The role of ontologies in biological and biomedical research: a functional perspective. Briefings in bioinformatics. 2015;16(6):1069–1080.
16. Munir K, Anjum MS. The use of ontologies for effective knowledge modelling and information retrieval. Applied Computing and Informatics. 2018;14(2):116–126.
17. Pang C. Computational methods for data discovery, harmonization and integration: Using lexical and semantic matching with an application to biobanking phenotypes [Phd Thesis]. University of Groningen; 2018. Available from: https://research.rug.nl/files/62157755/Complete_thesis.pdf.
18. Ashburner M, Ball CA, Blake JA, Botstein D, Butler H, Cherry JM, et al. Gene ontology: tool for the unification of biology. Nature genetics. 2000;25(1):25–29.
19. Consortium TGO. The Gene Ontology resource: enriching a GOld mine. Nucleic acids research. 2021;49(D1):D325–D334.
20. Giacomo GD, Lembo D, Lenzerini M, Poggi A, Rosati R. Using ontologies for semantic data integration. In: A Comprehensive Guide Through the Italian Database Research Over the Last 25 Years. Springer; 2018. p. 187–202.
21. Xiao G, Calvanese D, Kontchakov R, Lembo D, Poggi A, Rosati R, et al. Ontology-based data access: A survey. International Joint Conferences on Artificial Intelligence; 2018. .
22. Bienvenu M, Cate BT, Lutz C, Wolter F. Ontology-based data access: A study through disjunctive datalog, CSP, and MMSNP. ACM Transactions on Database Systems (TODS). 2014;39(4):1–44.
23. Toga AW, Bhatt P, Ashish N. Global data sharing in Alzheimer's disease research. Alzheimer disease and associated disorders. 2016;30(2):160.
24. Xiao C, Neu SC, Toga AW. Sharing of Alzheimer's Disease Research Data in the Global Alzheimer's Association Interactive Network. In: Alzheimer's Disease Drug Development: Research and Development Ecosystem. Cambridge University Press; 2022. p. 395–403.
25. Lovestone S, Consortium E. The European medical information framework: a novel ecosystem for sharing healthcare data across Europe. Learning health systems. 2020;4(2):e10214.
26. Bos I, Vos S, Vandenberghe R, Scheltens P, Engelborghs S, Frisoni G, et al. The EMIF-AD Multimodal Biomarker Discovery study: design, methods and cohort characteristics. Alzheimer's research & therapy. 2018;10(64).
27. Birkenbihl C, Salimi Y, Domingo-Fernándéz D, Lovestone S, Consortium A, Fröhlich H, et al. Evaluating the Alzheimer's disease data landscape. Alzheimer's & Dementia: Translational Research & Clinical Interventions. 2020;6(1):e12102.
28. Bauermeister S. Dementias Platform UK (DPUK): Facilitating Cross-Cohort Analysis in a Digital Age. In: 2020 Alzheimer's Association International Conference. ALZ; 2020. .
29. Weiner MW, Veitch DP, Miller MJ, Aisen PS, Albala B, Beckett LA, et al. Increasing participant diversity in AD research: Plans for digital screening, blood testing, and a community-engaged approach in the Alzheimer's Disease Neuroimaging Initiative 4. Alzheimer's & Dementia. 2023;19(1):307–317.
30. Mindt MR, Okonkwo O, Weiner MW, Veitch DP, Aisen P, Ashford M, et al. Improving generalizability and study design of Alzheimer's disease cohort studies in the United States by including under-represented populations. Alzheimer's & Dementia. 2022;.
31. Ashford MT, Camacho MR, Jin C, Eichenbaum J, Ulbricht A, Alaniz R, et al. Digital culturally tailored marketing for enrolling Latino participants in a web-based registry: Baseline metrics from the Brain Health Registry. Alzheimer's & Dementia. 2022;.
32. Dardiotis E, Kosmidis MH, Yannakoulia M, Hadjigeorgiou GM, Scarmeas N. The Hellenic Longitudinal Investigation of Aging and Diet (HELIAD): rationale, study design, and cohort description. Neuroepidemiology. 2014;43(1):9–14.
33. Anastasiou CA, Yannakoulia M, Kosmidis MH, Dardiotis E, Hadjigeorgiou GM, Sakka P, et al. Mediterranean diet and cognitive health: Initial results from the Hellenic Longitudinal Investigation of Ageing and Diet. PloS one. 2017;12(8):e0182048.
34. Ibanez A, Yokoyama JS, Possin KL, Matallana D, Lopera F, Nitrini R, et al. The multi-partner consortium to expand dementia research in Latin America (ReDLat): driving multicentric research and implementation science. Frontiers in neurology. 2021;12:631722.
35. Ciccarese P, Wu E, Wong G, Ocana M, Kinoshita J, Ruttenberg A, et al. The SWAN biomedical discourse ontology. Journal of biomedical informatics. 2008;41(5):739–751.
36. Malhotra A, Younesi E, Gündel M, Müller B, Heneka MT, Hofmann-Apitius M. ADO: A disease ontology representing the domain knowledge specific to Alzheimer's disease. Alzheimer's & dementia. 2014;10(2):238–246.
37. Henry V, Moszer I, Dameron O, Potier MC, Hofmann-Apitius M, Colliot O. Converting alzheimer's disease map into a heavyweight ontology: A formal network to integrate data. In: International Conference on Data Integration in the Life Sciences. Springer; 2018. p. 207–215.
38. Daly T, Bourdenx M, Henry V, Epelbaum S. Towards holistic theory and therapeutics of Alzheimer's disease: The AD-DROP ontology. Alzheimer's and Dementia. 2021;17.
39. Daly T, Henry V, Bourdenx M. From Association to Intervention: The Alzheimer's Disease-Associated Processes and Targets (ADAPT) Ontology. Journal of Alzheimer's Disease. 2023;.
40. Gomez-Valadés A, Martínez-Tomás R, Rincón-Zamorano M. Integrative Base Ontology for the Research Analysis of Alzheimerś Disease-Related Mild Cognitive Impairment. Frontiers Neuroinformatics. 2021;15.
41. Alzheimer's disease neuroimaging protocol (adni);. http://adni.loni.usc.edu/wp-content/themes/freshnews-dev-v2/documents/clinical/ADNI-1_Protocol.pdf.
42. Bechhofer S. In: LIU L, ÖZSU MT, editors. OWL: Web Ontology Language. Boston, MA: Springer US; 2009.

p. 2008–2009.
43. Prud'hommeaux E, Seaborne A. SPARQL Query Language for RDF - W3C Recommendation 15 January 2008. W3C; 2008.
44. Konys A. Knowledge repository of ontology learning tools from text. Procedia Computer Science. 2019;159:1614–1628.
45. Masri F. Automatic ontology learning from semi-structured data [Phd Thesis]. Czeck Technical University in Prague, Faculty of Information Technology; 2017. Available from: https://dspace.cvut.cz/bitstream/handle/10467/70147/F8-DP-2017-Masri-Filip-thesis.pdf?sequence=1&isAllowed=y.
46. Ben Mahria B, Chaker I, Zahi A. A novel approach for learning ontology from relational database: from the construction to the evaluation. Journal of Big Data. 2021;8(1):1–22.
47. Lovestone S, Francis P, Kloszewska I, Mecocci P, Simmons A, Soininen H, et al. AddNeuroMed—the European collaboration for the discovery of novel biomarkers for Alzheimer's disease. Annals of the New York Academy of Sciences. 2009;1180(1):36–46.
48. Genesereth MR, Nilsson NJ. Logical foundations of artificial intelligence. Morgan Kaufmann; 2012.
49. Gruber TR. A translation approach to portable ontology specifications. Knowledge acquisition. 1993;5(2):199–220.
50. Chaves ML, Godinho CC, Porto CS, Mansur L, Carthery-Goulart MT, Yassuda MS, et al. Cognitive, functional and behavioral assessment. Dementia & Neuropsychologia. 2011 09;5:153–166.
51. McGuinness DL, Van Harmelen F, et al. OWL web ontology language overview. W3C recommendation. 2004;10(10):2004.
52. de Coronado S, Tuttle MS, Solbrig HR. Using the UMLS Semantic Network to validate NCI Thesaurus structure and analyze its alignment with the OBO relations ontology. In: AMIA Annual Symposium Proceedings. vol. 2007. American Medical Informatics Association; 2007. p. 165.
53. Siemer S. Exploring the Apache Jena Framework. George August University Göttingen; 2019.

Figures

Figure 7 Family and Medical History.

Figure 8 Biospecimen Exam.

Figure 9 Genetic Exam.

Figure 10 3D Image Exam.
