# Supplementary figures and images for: An ontology-based approach for modelling and querying Alzheimer’s disease data
Source: BMC Med Inform Decis Mak. 2023 Aug 8;23:153. doi: 10.1186/s12911-023-02211-6 (PMC10408169; doi:10.1186/s12911-023-02211-6)

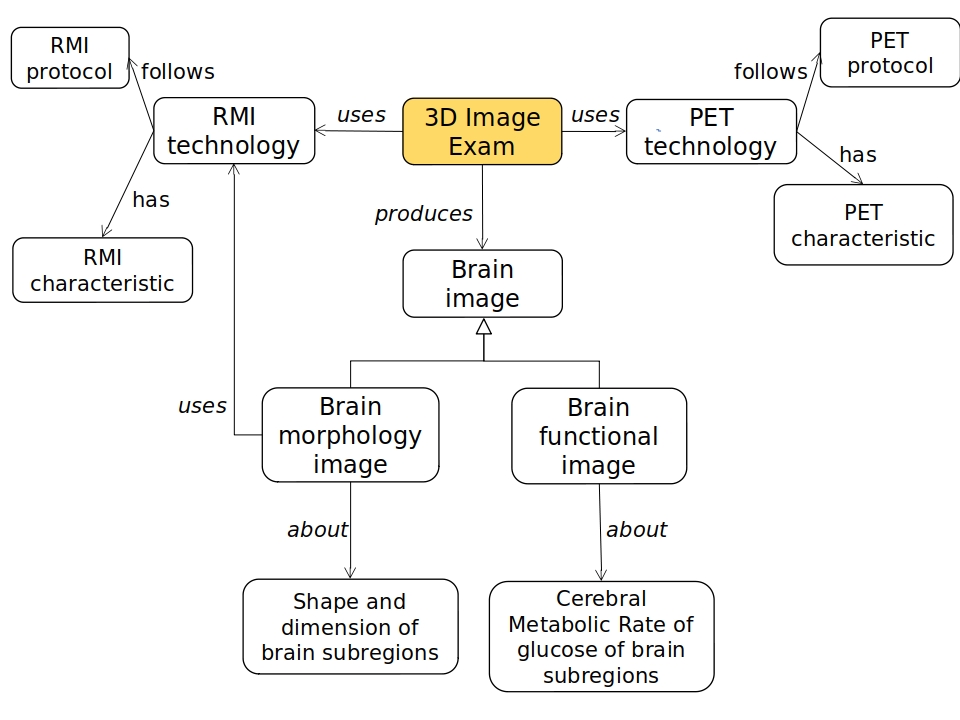

Supplement: Supplementary file 1 — Additional file 1. [file 12911_2023_2211_MOESM1_ESM.zip › Figure 10.jpg]

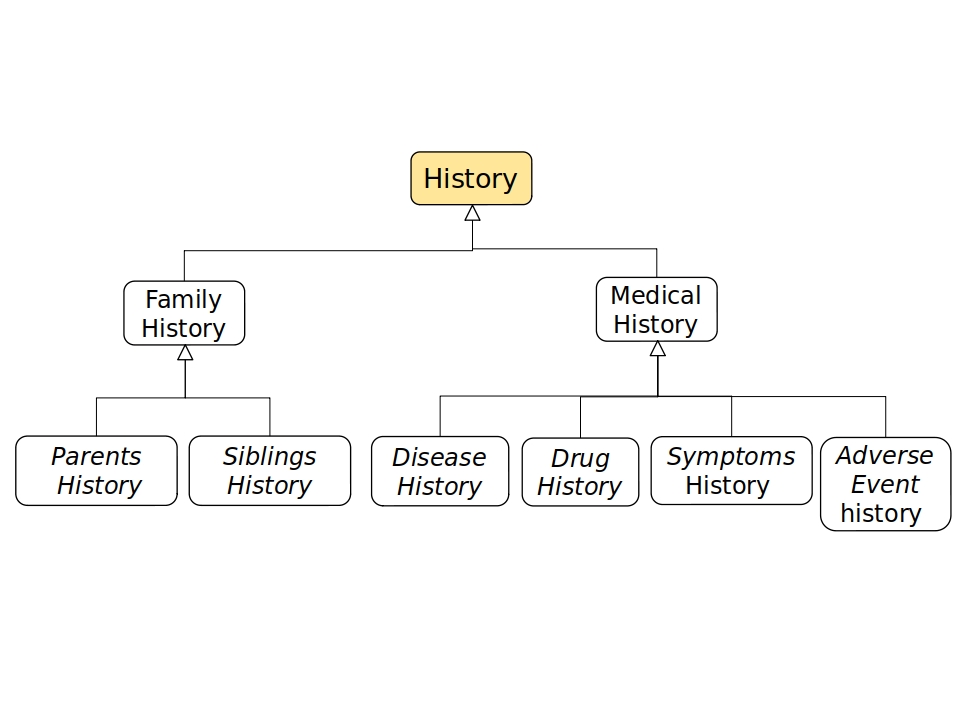

Supplement: Supplementary file 1 — Additional file 1. [file 12911_2023_2211_MOESM1_ESM.zip › Figure 7.jpg]

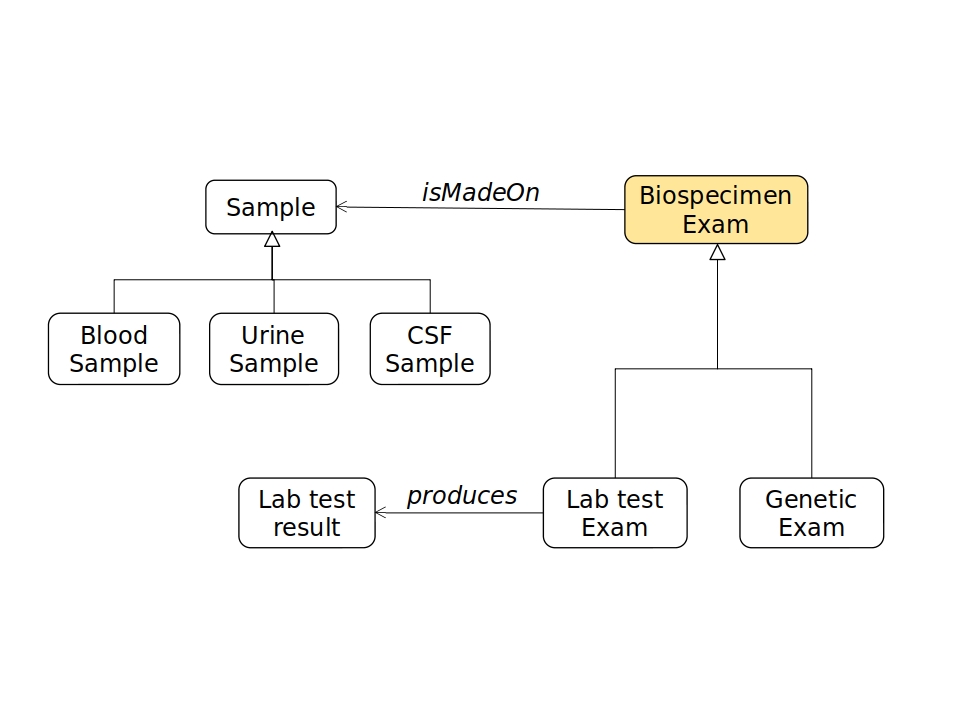

Supplement: Supplementary file 1 — Additional file 1. [file 12911_2023_2211_MOESM1_ESM.zip › Figure 8.jpg]

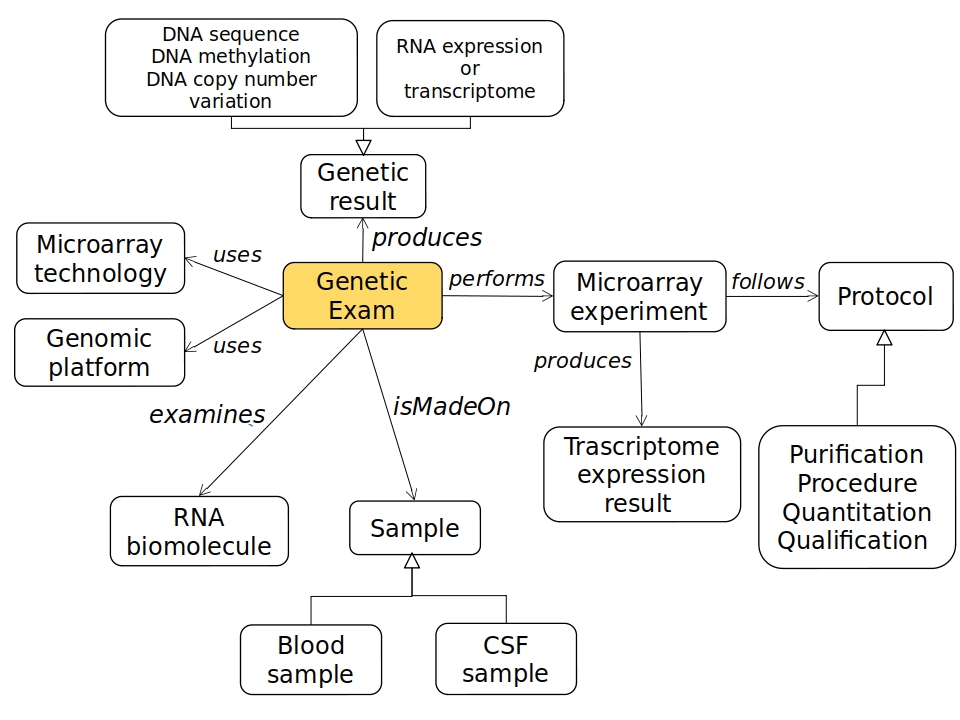

Supplement: Supplementary file 1 — Additional file 1. [file 12911_2023_2211_MOESM1_ESM.zip › Figure 9.jpg]
